# Supplementary figures and images for: Computational timeline reconstruction of the stories surrounding Trump: Story turbulence, narrative control, and collective chronopathy
Source: PLoS One. 2021 Dec 8;16(12):e0260592. doi: 10.1371/journal.pone.0260592 (PMC8654215; doi:10.1371/journal.pone.0260592)

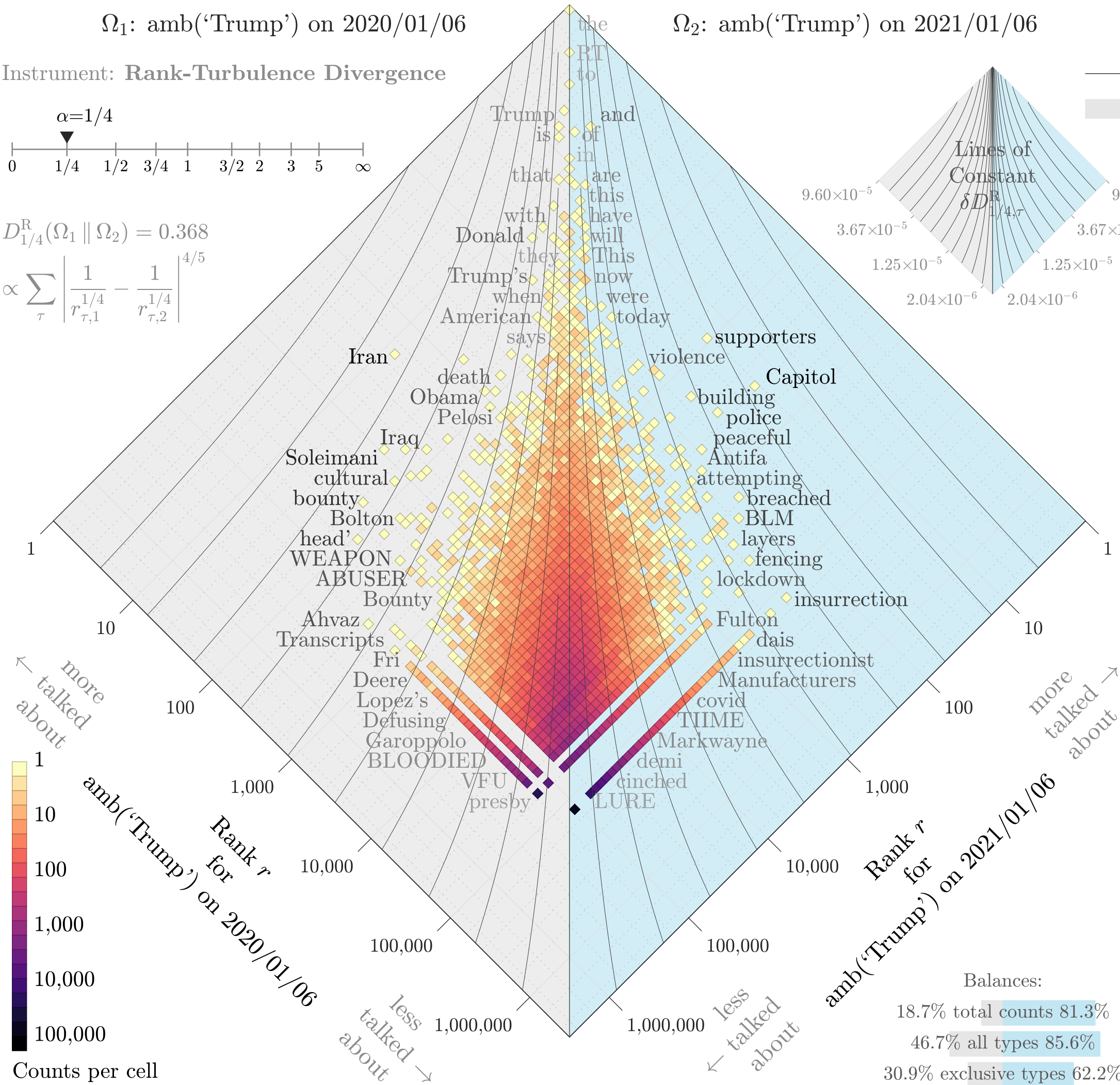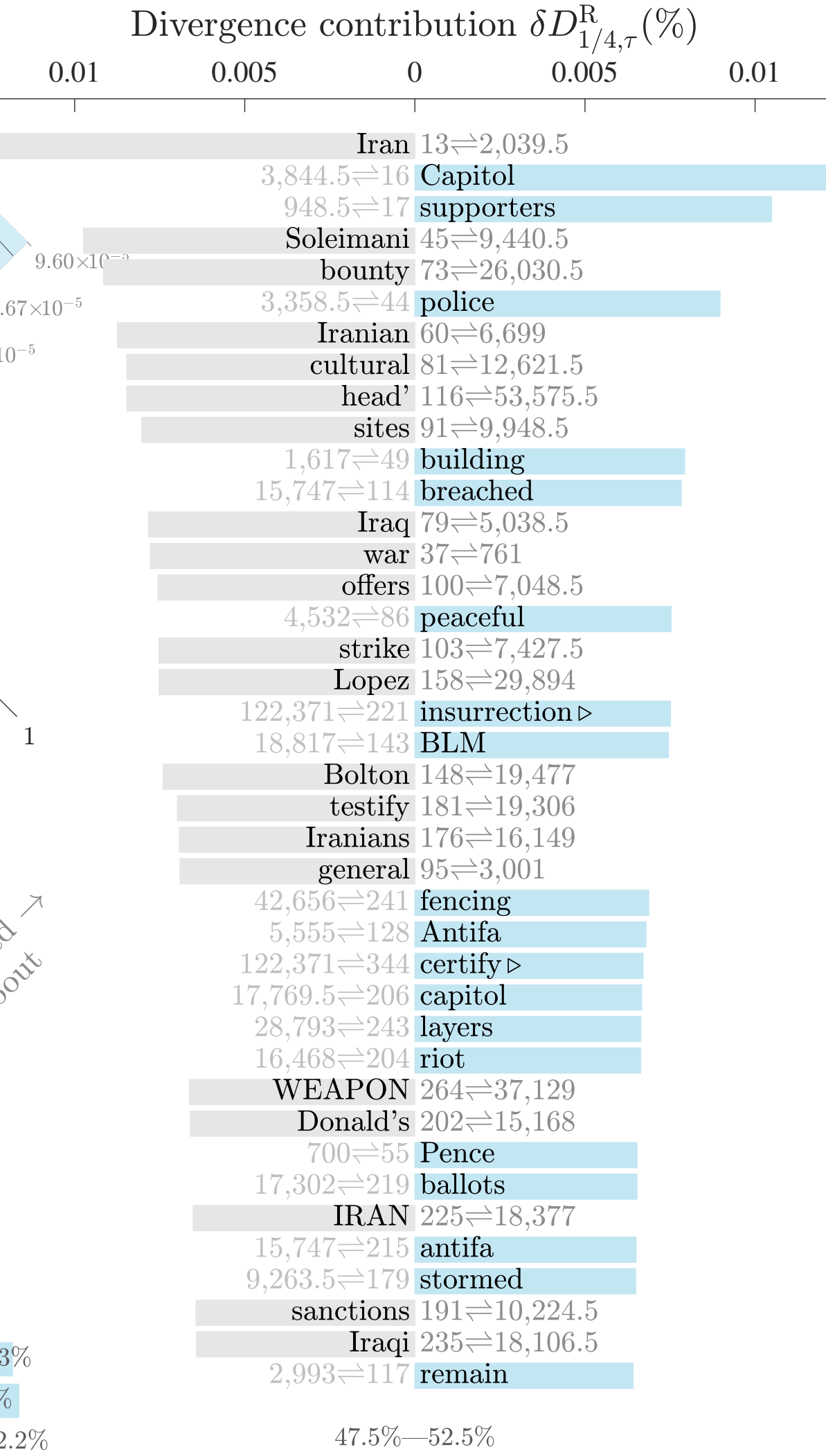

Supplement: S1 Fig — Our findings all build out from allotaxonometry: The principled measurement of the difference between the architectures of any two complex systems as represented by Zipf distributions [48]. For 2020/01/06 versus 2021/01/06, 1-grams on the first date reflect the aftermath of the assassination of Iranian military commander Qasem Soleimani by the United States on 2020/01/03, while 1-grams appearing on the second date revolve around the Capitol insurrection by Trump supporters. The rank-rank histogram on the left of the allotaxonograph displays the joint Zipf distribution, naturally laid out in double-logarithmic space. We generate the 1-gram ranking on the right of the allotaxonograph using rank-turbulence divergence (RTD) [37] with tuning parameter α = 1/4. The contour lines on the histogram provide guides for RTD showing a reasonable fit to the joint Zipf distribution’s form. Variations around α = 1/4 will not change the overall findings (i.e., the orderings of contributing 1-grams as well as the overall RTD score). See Ref. [37] for a full explanation of allotaxonometry and RTD. In Sec. 3.1 in the main paper, we use RTD at the year scale to determine narratively dominant 1-grams and 2-grams arising on the second of the two dates being compared. By comparing to a year ago, we are able to generate a background Zipf distribution that will help remove calendrical features as well as generically Twitter- and Trump-related 1-grams (e.g., ‘RT’ and ‘Donald’). While the allotaxonograph shows 1-grams on both dates, we emphasize that our focus is on the second date, in that we are seeking to determine the most important 1-grams of today. For 2020/01/06 versus 2021/01/06, looking at the ranked list on the right, we see that the top five 1-grams for the day of the Capitol insurrection are ‘Capitol’, ‘supporters’, ‘police’, ‘building’, and ‘breached’. The salience of ‘BLM’ (Black Lives Matter) and ‘Antifa’ point to the immediate confusion and disinformation surrounding the Capito [file pone.0260592.s001.pdf]
